# Assessment of the Learning Curve of Supercapsular Percutaneously Assisted Total Hip Arthroplasty in an Asian Population

**DOI:** 10.1155/2020/5180458

**Published:** 2020-09-07

**Authors:** Pengfei Lei, Zhan Liao, Jiang Peng, Guang Li, Qian Zhou, Xiao Xiao, Chunhua Yang

**Affiliations:** ^1^Department of Orthopedic Surgery, Hunan Engineering Research Center of Biomedical Metal and Ceramic Implants, Xiangya Hospital Central South University, Changsha, 410013 Hunan, China; ^2^Department of Orthopedics, The First Hospital of Changsha, Changsha, 410005 Hunan, China; ^3^Department of Orthopedics, The People's Hospital of Yichun City, 336400 Yichun, Jiangxi, China

## Abstract

The supercapsular percutaneously assisted total hip (SuperPATH) approach is a microinvasive approach that was developed to minimize surgical disruption of soft tissue during routine total hip arthroplasty (THA). This study was aimed at assessing early outcomes and learning curves of the SuperPATH approach in one Chinese hospital's experience. Early outcomes of the first consecutive 78 SuperPATH cases (80 hips) performed by the same surgeon were evaluated. The patients were divided into 4 groups according to the surgical order. The incision, intraoperative blood loss, hospital stay, Harris hip score, and complication occurrence in each group were evaluated. Learning curves were assessed using operative time and intraoperative blood loss as surrogates. The operation time and intraoperative blood loss of groups A and B were more than those of groups C and D, and the difference was statistically significant (*P* < 0.05); however, there was no statistically significant difference between the two groups (group A vs. group B, *P* = 0.426; group A vs. group B, *P* = 0.426). There was no statistically significant difference in terms of incision length and hospital stay, and Harris hip score at the last follow-up was increased with statistically significant difference when compared with that preoperatively among the 4 groups. One case of periprosthetic fracture occurred in group A. No other complication, such as joint dislocation, sciatic nerve injury, prosthesis loosening, periprosthetic infection, and deep vein thromboembolism, occurred in the 4 groups. In summary, for surgeons who are familiar with the standard posterolateral approach, they could achieve more familiarity with SuperPATH after 40 cases of surgery.

## 1. Introduction

Total hip arthroplasty (THA) is an effective treatment for hip osteoarthritis and other disorders like femoral neck fracture, femoral head necrosis, and acetabular dysplasia [1]. There are 3 main classical approaches to the hip for THA: posterolateral (Kocher-Langenbeck), anterolateral (Watson-Jones), and anterior (Smith-Peterson). Large clinical studies have shown excellent results from all 3 methods of THA [2]. Nonetheless, pain control, length of postoperative rehabilitation, and complications from the surgical approach [3] have led surgeons to modify those traditional approaches to become more tissue-friendly. Commonly cited modifications currently include the direct anterior approach (DAA), Anterior supine intermuscular approach (ASI or OCM), and modified miniposterior approach. Each of these minimally invasive approaches has been shown to be safe and effective, if performed outside of its “learning curve” [1]. Each learning curve has been reported to be different based on approach, author, and patient population [4].

Additional modifications have aimed at further reducing tissue trauma, as well as streamlining the learning curve and surgeon experience. One such heavily modified posterolateral approach (now known as microposterior or microsuperior approach), the supercapsular percutaneously assisted total hip (SuperPATH) approach, has been carried out in many countries [5]. It was developed by Dr. Chow et al. [6] and combines elements of previous work by Dr. Penenberg et al. and Dr. Murphy and Tannast from the PATH and SuperCap approaches [7, 8]. This transgluteal technique uses the interval between the gluteus minimus and piriformis muscles, is aimed at sparing all hip girdle muscles, and maintains the mechanical and neurologic integrity of the joint capsule by using only an inline incision [8, 9]. Compared with the standard posterolateral approach, this technique has the potential advantages of smaller incision, less intraoperative bleeding, shorter postoperative recovery, and fewer complications [5]. With the advantages aforementioned, SuperPATH seems to be very suitable for Asian patients as aging is becoming the heavy burden for this region and society.

However, as with any surgical technique, SuperPATH has an associated learning curve with its adoption [5, 6]. Previous studies have defined learning curve with other approaches as a duration of cases with a longer surgical time, higher surgical bleeding, higher component malpositioning rates, and higher complication rates [5, 6]. Previous publications for SuperPATH have shown a clear learning curve with respect to operative time, but with no influence on complication or component positioning [6, 10]. The absence of complications or safety issues during the learning curve was associated with the ease and readiness of conversion to a miniposterior approach and further to the standard posterior approach [10, 11]. If any difficulty or complication is encountered during the SuperPATH approach, it can quickly be extended to allow the surgery to be performed more easily. Despite these earlier studies regarding the safety of the learning curve of SuperPATH, to our knowledge, no study has been done to date where there are pressures to avoid conversion to a posterior approach. If a posterior approach conversion is to be avoided, an alternative, more restricted learning curve for SuperPATH can be shown.

Unique to many Chinese practices, there are significant cultural patient and physician pressures to “stay the course” once the decision has been made to proceed with a surgical strategy. It is culturally uncommon to begin a surgery as SuperPATH and readily convert it to the posterior approach. Because of this, Chinese data is uniquely positioned to show another side of the SuperPATH, where the surgeon does not alter the procedure for safety. To our knowledge, there has been no study reporting the learning curve of the SuperPATH approach in THA for Chinese patients. In this study, the learning curves for the SuperPATH cohorts were assessed using operative time and intraoperative blood loss as a surrogates. All surgeries that started as SuperPATH were completed as SuperPATH procedures without conversion to the posterior approach. These cohorts were subdivided into groups of four consecutive cases. Mean operative time and intraoperative blood loss for each subgroup were calculated and compared using the correlation coefficient to determine whether operative time and intraoperative blood loss decreased as the surgeon gained experience with the technique.

## 2. Methods

### 2.1. Patients

This single-center retrospective study assessed 78 total hip arthroplasty patients (80 hips) at The First Hospital of Changsha in June 2017. In the chronological order of the operation, patients were divided into 3 groups: group A (*n* = 20, 20 hips), group B (*n* = 19, 20 hips), group C (*n* = 19, 20 hips), and group D (*n* = 20, 20 hips). All surgeries were performed by a single senior orthopedic surgeon. Preoperatively, all patients routinely underwent preadmission assessment, including radiograph of the pelvis and hip joint, blood tests, electrocardiogram, and anesthesiologist assessments. Inclusion criteria were as follows: (1) patients with body mass index (BMI) < 40 kg/m^2^; (2) patients without serious functional deformity; (3) patients without the surgical history in the affected side hip joint; and (4) patients undergoing primary THA. Exclusion criteria were as follows: (1) patients with congenital dislocation of the hip joint (grade 3 or grade 4 according to the Crowe classification); (2) patients with body mass index (BMI)≧40 kg/m^2^; (3) patients with severe heart and lung dysfunction; (4) patients with stiff joints; and (5) patients with overt infection in the hip joint or other contraindications for THA. This study has been approved by the Medical Ethics Committee of the First Hospital of Changsha (KL-2020007), and written informed consent was obtained from each patient before the surgical procedures.

### 2.2. Surgical Technique

Preoperatively, the standardized anteroposterior radiograph of the pelvis and hip joint of the operative leg was taken, and then, templating was performed according to the radiograph. Next, the position of the osteotomy and the length of the femoral neck were predicted. An appropriate type of prosthesis was selected according to the radiograph. The patient was positioned in standard lateral decubitus assisted by a patented hip surgery positioning device (Dr. Lei's Table for Hip and Knee Surgery, Chengdu medical device registration number 20180087, Sichuan, China), with the operative hip flexed in 45° and 10 to 15° of internal rotation to position the greater trochanter upward. The foot of the operative leg was elevated on a surgical tray. Following standard aseptic preparation and draping of the operative site, a skin incision was made from the tip of the greater trochanter to the fascia of the gluteus maximus with a length of 6 to 8 cm and in line with the femoral axis. The gluteus maximus was carefully split by wing tip elevators, and then, a Cobb elevator was placed under the gluteus medius. Next, a Cobb elevator was replaced with a blunt Hohmann retractor and the Hohmann retractor was placed in the gap between the gluteus medius and the gluteus minimus to protect the gluteus medius and pull the gluteus medius forward.

The hip joint was externally rotated to a more neutral position. Another Cobb elevator was placed between the piriformis tendon and the gluteus minimus and then also replaced with a blunt Hohmann retractor. This Hohmann retractor was placed between the posterior joint capsule and external rotators, and the piriformis was retracted to expose the hip capsule. The capsule was then incised along the path of the skin incision. The trochanteric fossa was incised with electrocautery to ensure haemostasis at the base of the femoral neck. The acetabular rim was separated from the joint capsule, and the incision was extended 1 cm to expose the piriform fossa, the tip of greater trochanter, and the anterior femoral neck.

The operative leg was rotated to a more neutral position to expose the saddle of the femoral neck. An entry reamer was used to open the femoral canal through the trochanteric fossa, and then, a metaphyseal reamer was used to expand the incision. The appropriate anteversion angle at the femoral neck and head was kept to insert the intramedullary broach and increase the size of intramedullary broaching to complete the preparation of medullary cavity with the suitable size. Then, to remove the handle, the femoral neck was cut off using a narrow oscillating saw blade along the top of the intramedullary broach. In removing the femoral head, two stirling needles were drilled into it for lever force. If necessary, the femoral head could be cut into smaller pieces with osteotome.

A Zelpi retractor was placed beneath the acetabular margin to retract the capsule proximally, and a Romanelli retractor placed for distal retraction. Under direct vision, all remaining soft tissues in the acetabulum and labrum were removed. With the assist of a guide device, a cannula was inserted through a 1 cm skin incision to the main incision. The appropriate-size acetabular reamer was placed into the acetabulum by the main incision, the reamer holder passed through the cannula tube, and matched with the reamer in situ. After multiple graded reaming of the acetabulum, the appropriate-sized cementless acetabular cup (MicroPort Orthopedics Inc., Arlington, TN, USA) was implanted, and then, 2 screws were generally placed for primary fixation. The high cross-linked polyethylene liner was inserted and locked using an impactor through the cannula. The appropriately sized femoral head and modular neck and stem components were trialled. The range of motion and leg length were checked, and the stability of the joint was evaluated. After removing the trial components, the cementless modular femoral stem prosthesis and the femoral head (MicroPort Orthopedics Inc., Arlington, TN, USA) were implanted. Finally, the capsule and gluteal fascia were sutured, and the skin incision was closed layer by layer.

### 2.3. Outcome and Clinical Assessment

Harris hip score was used to assess the hip function recovery [12]. The higher scores indicate better hip function. Besides, operative time, peroperative bleeding, length of surgical incision, hospital stay, and adverse events were recorded to evaluate the early outcomes. The learning curve was assessed using operative time and peroperative bleeding as the surrogate.

### 2.4. Postoperative Management

According to the postoperative orthopaedic venous thromboembolism prevention principle [13], as per our hospital's standard, patients were routinely offered subcutaneous injection of low molecular heparin calcium (5000 IU, once a day) for anticoagulant and also offered preventive anti-infection and wound dressing processing after 24-hour operation. Within the first day after surgery, patients were allowed to perform hip joint flexion and extension exercises and walking with the help of ambulatory assist.

### 2.5. Statistical Analysis

Statistical analysis was performed with SPSS Statistics 19.0 (SPSS Inc., Chicago, Illinois, USA). Quantitative data were described by mean ± standard deviation (SD). Qualitative data were described by number or percentage. The normality of the data distribution was tested with the Kolmogorov-Smirnov test. Qualitative variables were analyzed by using the *χ*^2^ test. Paired sample *t*-test was used to compare the Harris score of the hip joint before and after operation. Analysis of variance was used to compare the operative time, preoperative bleeding, length of surgical incision, and hospital stay among groups. Differences were considered statistically significant when *P* < 0.05.

### 2.6. Results

**Figure 1:**
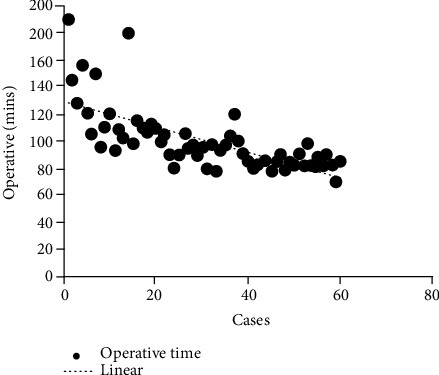
Learning curve of the SuperPATH approach with operative time as a parameter, showing that approximately 40 cases reached asymptotes.

**Figure 2:**
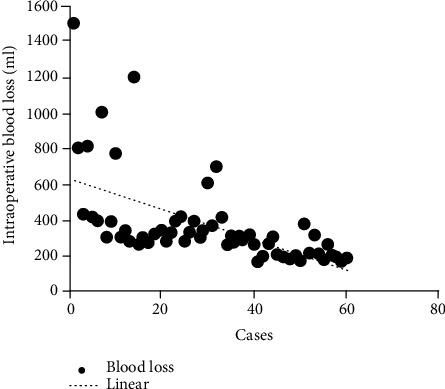
Learning curve of the SuperPATH approach with intraoperative blood loss as a parameter, showing that approximately 40 cases reached asymptotes.

No significant difference was identified between the four groups in terms of age, gender, BMI, or diagnosis result (Table 1). The operation time of group A was longer than that of group B, and the difference was statistically significant (*P* ≤ 0.001), and it was significantly longer than that of group C or D (group A vs. group C, *P* ≤ 0.001; group A vs. group D, *P* ≤ 0.001; group B vs. group C, *P* ≤ 0.001; group B vs. group D, *P* ≤ 0.001); however, there was no statistically significant difference between the two groups (group C vs. group D, *P* = 0.426). Intraoperative blood loss of group A was more than that of group B, and it was significantly more than that of group C or D (group A vs. group C, *P* ≤ 0.001; group A vs. group D, *P* ≤ 0.001; group B vs. group C, *P* = 0.009; group B vs. group D, *P* = 0.003); however, there was no statistically significant difference between the two groups (group C vs. group D, *P* = 0.716). There was no statistically significant difference in terms of incision length and hospital stay among the 4 groups. (Table 2) The intraoperative blood loss and operation time of the patients in the 4 groups were basically flat in the trend line after 40 hips (Figures 1 and 2).

**Figure 3:**
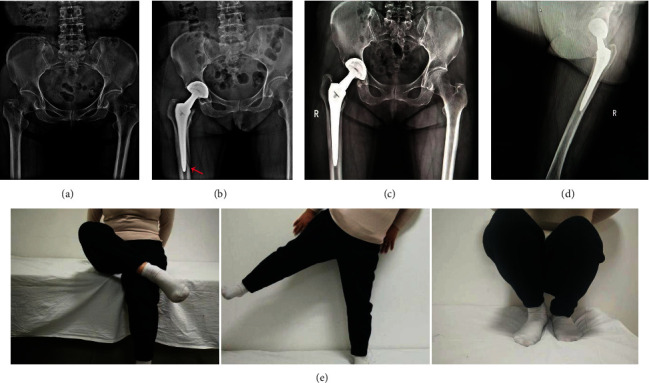
(a) Preoperative X-ray of a 59-year-old female patient with right femoral neck fracture. (b) The results of X-ray showed that intraoperative periprosthetic fracture occurred (red arrow). (c) The results of X-ray 3 months after surgery showed that the reconstruction of the leg length and offset of hip joint was good. (d) X-ray at 6 months after surgery. (e) The pictures showed good function and stable hip joint.

**Figure 4:**
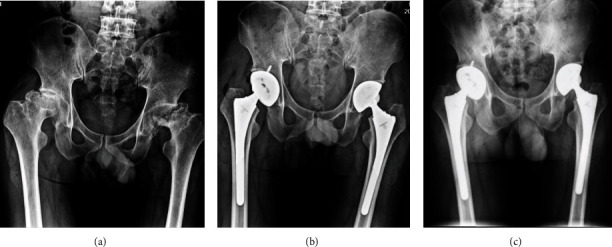
(a) Preoperative X-ray of a 69-year-old male patient with bilateral femoral head necrosis ARCO IV (HIV patients) who underwent bilateral SuperPATH approach. (b) The results of X-ray 1 week after surgery showed that the reconstruction of the length and eccentricity of both lower limbs was good. (c) X-ray at 6 months after surgery.

Two typical cases are shown in Figures 3 and 4. One case of intraoperative periprosthetic fracture occurred in group A, due to the small femoral bone marrow cavity and poor preoperative planning, resulting in the femoral shaft splitting with the smallest prosthesis insertion (Figure 3). No other complication, such as dislocation, sciatic nerve injury, prosthesis loosening, periprosthetic infection, and deep vein thromboembolism, occurred in patients of the 4 groups.

For all patients, the mean follow-up time was 10.5 months (range 6-12 months). As Table 3 has shown, in the four groups, the Harris hip score at the last follow-up was increased with statistically significant difference when compared with that preoperatively: group A was increased from 32.39 ± 14.66 to 92.33 ± 4.75 (*t* = 18.942, *P* ≤ 0.001), group B was increased from 30.25 ± 18.79 to 94.13 ± 4.25 (*t* = 16.916, *P* ≤ 0.001), group C was increased from 31.53 ± 20.13 to 94.25 ± 3.10 (*t* = 14.907, *P* ≤ 0.001), and group D was increased from 31.61 ± 18.88 to 94.30 ± 2.61, (*t* = 14.688, *P* ≤ 0.001).

## 3. Discussion

The SuperPATH approach is a new progress in minimally invasive THA; it combines the advantages of the SuperCap approach and PATH approach [6, 7, 14, 15]. It can significantly reduce the incidence of complications in such patients, allow rapid rehabilitation, and reduce the use of special traction beds and equipment. This is of great significance for elderly patients with femoral neck fractures, and it is an easier and safer method for surgeons, which is worthy of clinical application. At the same time, with the development and prosperity of minimally invasive surgery, the learning curve of surgeons' proficiency in new technologies in the field of minimally invasive surgery has become the focus of clinical research [5].

The learning curve is usually used to evaluate the difficulty of a new minimally invasive surgery; the shorter learning curve indicates that the technique is easier to master. Its descriptive indicators mainly include operation time, bleeding volume, conversion rate, complication occurrence, length of hospital stay, and surgical efficacy [16, 17]. Previously, Rasuli and Gofton [5] assessed early outcomes and learning curves of the 49 consecutive cases of the PATH approach and 50 cases of the SuperPATH approach; the results showed that the PATH group operative time reached a plateau by case 40, but the SuperPATH operative time continued to decrease by case 50. However, in our study, we retrospectively analyzed the surgical results of 78 Asian patients (80 hips) who underwent the SuperPATH approach by the same surgeon, the results showed that after 40 cases of SuperPATH, the intraoperative blood loss was flat, which might mean that the learning curve of the SuperPATH approach was about 40 cases and suggest that this technique could be generalized to orthopedic surgeons adopting it.

The problems we encountered are mainly at the early stage of the learning curve, such as not proficient in proprietary surgical instruments, long operative time, larger intraoperative blood loss, difficulty with retaining the external rotators via a small incision, limited intraoperative vision and operating space, and higher risk of early complications. In this study, in the early stage of the learning curve, we encountered some problems as follows. (1) The unsatisfactory placement of the prosthesis was due to the fact that during the operation, the assistant used the bone hook to pull the femur forward, which caused the pelvis to lean forward, resulting in a small anteversion angle of acetabular lateral prosthesis. For this, we could increase 8° to 15° of anteversion at the lateral femur using the combination handle, so that an ideal combined anteversion could be achieved. The safe zone, which has been shown to be associated with a lower postoperative dislocation rate, is defined by a cup anteversion of 5° to 25° and abduction of 30° to 50° [18]. Therefore, it was recommended that in the early learning curve, beginners choose the appropriate combination handle rather than the integrated handle, which can increase the fault tolerance rate. (2) One case of periprosthetic fracture occurred in group A, due to the short stature, smaller femoral bone marrow cavity, and poor preoperative planning, resulting in the smallest prosthesis in the operation that could not be placed in the right place. Gofton et al. [10] reported that the incidence of fractures around the prosthesis with the SuperPATH approach was 0.8%, which reminded us to be cautious at the early stage of the learning curve; the size of prosthesis should be estimated preoperatively; and if necessary, the size and location of prosthesis should be confirmed by intraoperative multiperspective. (3) Acetabular screw, drilling, and screw placement were difficult. In the case of good muscle relaxation during the operation, the direction of the cannula could be adjusted by moving the hip joint to make it consistent with the direction of the pinhole in the cup. Murphy et al. [19] believed that soft tissue protection technology of the incision from the joint capsule above, because the acetabulum needs to be exposed vertically from the side to the middle, also makes screw implantation more difficult than acetabulum cup implantation, especially for obese people.

How to improve the surgical effect and reduce complications as soon as possible within the early learning curve and accelerate the rise of the learning curve is the unremitting goal of every surgeon. In the early implementation of the SuperPATH approach, we should pay attention on the following aspects: (1) experienced in hip replacement surgery; (2) fixed cooperative surgical team in the OR; (3) intraoperative controlled hypotension of tranexamic acid and satisfactory muscle relaxant effect [20]; (4) patient selection; and (5) detailed preoperative plan, including a detailed history based on preoperative imaging measurements, the location of the osteotomy, and the length of the femoral neck were estimated, and the appropriate type and size of prosthesis were selected. Our results were different from the rest of the world regarding SuperPATH, and this was likely due to lack of conversion readiness. We recommend that the surgeons more easily convert surgery to the posterior approach to avoid pitfalls during the learning curve.

Limitations of this study include the lack of randomization. However, randomization may have inappropriately lengthened the learning curve by increasing the time interval. The absence of a control group is also a weakness of this study, and further studies are required to confirm the result. Other limitations of this study include small sample size, lack of long-term follow-up, and lack of functional results. Considering the factors of education level and participation level of elderly patients in China, the simple score method—Harris score—was chosen. In the future, a larger sample size will be collected to confirm the conclusions of this study, with the updated score methods.

## 4. Conclusion

In generally, for surgeons who are familiar with the standard posterolateral approach, they could achieve more familiarity with SuperPATH after 40 cases of surgery. In order to improve the surgical effect, reduce complications, and shorten the learning curve process as much as possible, we suggest that appropriate cases should be selected at the early stage or convert surgery to the posterior approach, if necessary with a detailed preoperative plan and intraoperative fluoroscopy in multidimensions.

## Figures and Tables

**Table 1:** Comparison of baseline characteristics of patients in four groups.

Characteristics	Group A (*n* = 20, 20 hips)	Group B (*n* = 19, 20 hips)	Group C (*n* = 19, 20 hips)	Group D (*n* = 20, 20 hips)	*P*
Sex (male/female)	8/12	8/11	6/13	8/12	0.443
Mean age (years)	60.40 ± 9.60	62.40 ± 15.62	69.35 ± 10.04	65.75 ± 11.42	0.98
BMI (kg/m^2^)	24.26 ± 2.84	24.21 ± 2.63	24.43 ± 3.13	25.34 ± 2.80	0.57
Diagnosis (femoral neck fracture/femoral head necrosis), case	9/11	12/8	12/8	10/10	0.715

**Table 2:** Comparison of clinical indicators of learning curve in four groups.

Characteristics	Group A (*n* = 20, 20 hips)	Group B (*n* = 19, 20 hips)	Group C (*n* = 19, 20 hips)	Group D (*n* = 20, 20 hips)	*P*
Operative time (mins)	122.65 ± 27.45	100.40 ± 8.65	82.85 ± 5.44	79.00 ± 8.22	≤0.001
Intraoperative blood loss (ml)	522.00 ± 346.97	359.00 ± 111.96	201.50 ± 60.80	180.00 ± 47.46	≤0.001
Length of incision (cm)	8.01 ± 0.77	7.81 ± 0.22	7.69 ± 0.49	7.71 ± 0.50	0.224
Hospital stay (days)	12.00 ± 4.72	11.60 ± 4.27	11.60 ± 4.21	10.58 ± 4.16	0.860

**Table 3:** Clinical efficacy evaluation of the 4 groups of patients before and at the last follow-up.

Group	Harris hip score		*t*	*P*
	Preoperative	Last follow-up		
Group A (*n* = 20, 20 hips)	32.39 ± 14.66	92.33 ± 4.75	18.942	≤0.001
Group B (*n* = 19, 20 hips)	30.25 ± 18.79	94.13 ± 4.25	16.916	≤0.001
Group C (*n* = 19, 20 hips)	31.53 ± 20.13	94.25 ± 3.10	14.907	≤0.001
Group D (*n* = 20, 20 hips)	31.61 ± 18.88	94.30 ± 2.61	14.688	≤0.001

## Data Availability

The data used to support the findings of this study are available from the corresponding author upon request.
